# Pediatric Hematology–Oncology Provider Attitudes and Beliefs About the Use of Acupuncture for Their Patients

**DOI:** 10.3390/children12080961

**Published:** 2025-07-22

**Authors:** Holly L. Spraker-Perlman, Kenneth M. Busby, Amy Ly, Maggi Meyer, Justin N. Baker, Deena R. Levine

**Affiliations:** 1 Department of Pediatrics, Divisions of Pediatric Hematology-Oncology & Pediatric Palliative Care, University of Utah, Primary Children’s Hospital, Salt Lake City, UT 84113, USA; 2 Department of Pediatrics, Stanford University, Palo Alto, CA 94304, USA; kmbusby@stanford.edu; 3 Department of Pediatric Medicine, Division of Pediatric Neurology, St. Jude Children’s Research Hospital, Memphis, TN 38105, USA; amy.ly@stjude.org; 4 Department of Rehabilitation Medicine, St. Jude Children’s Research Hospital, Memphis, TN 38105, USA; maggi.meyer@stjude.org; 5 Division of Quality of Life and Pediatric Palliative Care, Department of Pediatrics, Stanford University School of Medicine, Palo Alto, CA 94305, USA; jusbaker@stanford.edu; 6 Department of Oncology, Division of Quality of Life and Palliative Care, St. Jude Children’s Research Hospital, Memphis, TN 38105, USA; deena.levine@stjude.org

**Keywords:** pediatrics, children, oncology, cancer, acupuncture, survey, needs assessment, integrative medicine

## Abstract

**Background/Objectives**: Children with cancer suffer due to the underlying disease and prescribed cancer-directed therapies, and non-pharmacologic modalities may offer improved symptom control without additional medications. We sought to elicit knowledge, attitudes, and beliefs of Pediatric Hematology Oncology (PHO) providers surrounding the incorporation of acupuncture for symptom management for their patients. **Methods**: A cross-sectional survey instrument was created, formatted, and delivered to physicians and advanced practice providers (APPs) at a single US pediatric cancer center. Survey responses were summarized by descriptive statistics. **Results**: A total of 78 PHO clinicians participated (response rate 29%). Most participants were interested in learning more about acupuncture (*n* = 42, 56.0%), yet rarely (*n* = 17, 22.7%) or never (*n* = 46, 61.3%) recommend acupuncture to patients. Most (*n* = 51, 73.9%) noted that they would support institutional development of an acupuncture program. Over half (*n* = 37, 52.2%) indicated their threshold for minimum hematologic indices for acupuncture includes a platelet count greater than 20,000 and absolute neutrophil count (ANC) greater than 500 (*n* = 37, 54.4%). Approximately two-thirds (*n* = 52, 66.7%) of participants noted that acupuncture could improve their patient’s quality of life, and most (*n* = 46, 67.6%) were not worried about harm. **Conclusions**: Acupuncture for symptom management is an evidenced-based, guideline-concordant recommendation for adults with cancer, but robust data in the pediatric oncology population are lacking. PHO providers do not routinely recommend acupuncture for patients but note that it may improve quality of life. Given their high symptom burden, rigorous studies of non-pharmacologic strategies for pediatric symptom management are vital. Acupuncture should be examined as a potential beneficial adjunct.

## 1. Introduction

Acupuncture is one component of Traditional Chinese Medicine whereby thin, sterile needles are inserted into specific body areas to help with symptom relief and to promote wellness [1]. Acupuncture can be combined with acupressure, when acupoints are stimulated with practitioner hands or cups, avoiding needles completely. Acupuncture is used frequently in adults with cancer and now is recommended in several national guidelines for oncology symptom management. The National Comprehensive Cancer Network (NCCN), for instance, recommends consideration of acupuncture in adult survivorship, particularly for fatigue, neuropathic pain, arthralgias, myalgias, myofascial pain, nausea, and vomiting [2]. The Society for Integrative Oncology (SIO) and the American Society of Clinical Oncology (ASCO) joint guidelines recommend acupuncture for adult patients with generalized cancer pain or musculoskeletal pain, though they deemed that there was insufficient or inconclusive evidence to make recommendations for pediatric patients [3]. Studies on acupuncture use for adult cancer patients are plentiful, but of lower quality than desired. However, recent data show that acupuncture may improve chemotherapy-induced peripheral neuropathy symptoms and quality of life in cancer survivors with persistent symptoms [4]. The most rigorous data for the use of acupuncture for cancer patients is for chemotherapy-induced nausea and vomiting (CINV) and endocrine symptoms (primarily “hot flashes”) associated with breast and prostate cancer therapy [5,6,7].

Quality data on the use of acupuncture in children is less rigorous than desired. However, several recent meta-analyses showed that the use of acupuncture for CINV was promising despite the lower quality of evidence [8,9,10]. Though data are limited regarding the use of acupuncture specifically for pediatric oncology patients, available evidence supports the use of acupuncture for pediatric pain and nausea outside of the oncology population. Acupuncture has been utilized to decrease pain intensity and Pain Dimensions scores for children with chronic pain, has been effective in decreasing numerical rating scale for pain in patients with Pediatric Complex Regional Pain Syndrome (CRPS), and has been effective in adolescents with endometriosis-related pelvic pain [11,12,13]. Several randomized clinical trials have found that acupuncture may be effective for children with colic pain, procedure anxiety, headache, and dysmenorrhea [12]. Acupuncture, in conjunction with conventional antiemetics, has proven beneficial for postoperative nausea in pediatric patients [14]. Despite evidence for the use of acupuncture for symptom management in a general pediatric population, any intervention for children undergoing cancer therapy must be weighed carefully. The risks of acupuncture may be amplified due to frequent immunocompromise and potential for thrombocytopenia, though the rewards of symptom mitigation without additional medications or side effects may outweigh these risks.

Acupuncture utilization data for pediatric oncology patients is limited in the literature to single-center publications [15]. It is unclear how often acupuncture is incorporated into pediatric cancer care nationally, but based on the amount of published data, it is less well integrated than in adult oncology settings. Facilitating the introduction of acupuncture to pediatric patients and their families may start with healthcare providers. Pediatric hematology–oncology (PHO) physicians are responsible for recognizing interventions that may provide benefit for their patients in a risk-adapted fashion and making recommendations for use. For instance, a previous description of surveyed physicians from 2009 found that 45% of physicians believed that acupuncture may improve quality of life for their patients [16]. Ideally, all safe and potentially beneficial interventions should be considered and discussed in a family-centered way to provide the best care for patients. We sought to assess the knowledge, attitudes, and underlying beliefs of PHO providers surrounding the use of acupuncture in their patients to identify barriers and facilitators to recommendation.

## 2. Materials and Methods

A comprehensive review of the literature confirmed a lack of validated instruments to assess knowledge and attitudes toward acupuncture in PHO physicians and advanced practice providers (APPs). Metrics from prior studies were combined with newly developed pediatric- and oncology-specific questions to create a cross-sectional survey instrument assessing provider knowledge, attitudes, and beliefs about integrative modalities for PHO patients as previously described [17,18]. The finalized questions were formatted in the DataSTAT electronic survey platform (Ann Arbor, MI, USA). Surveys were delivered to physicians (MD/DO) and advanced practice providers (APPs; nurse practitioners and physician assistants) at St. Jude Children’s Research Hospital. Participants acknowledged consent for de-identified data use. The institutional review board declared this project exempt from full review.

Survey responses were collected and summarized by descriptive statistics. Associations between selected questions were calculated using Chi-square or Fisher’s exact tests. Adjustment for multiple tests required false-discovery-rate correction. Statistical analyses were conducted using SAS 9.4 (SAS Institute, Cary, NC, USA). Statistical analysis using Chi-squared testing assured that the physician and APP cohorts were not different in terms of their personal use of integrative modalities, beliefs about integrative medicine (IM) education, or communication with patients surrounding integrative therapies. As such, physicians and APPs were analyzed as one cohort (Supplemental Data Table S1).

## 3. Results

**Figure 1:**
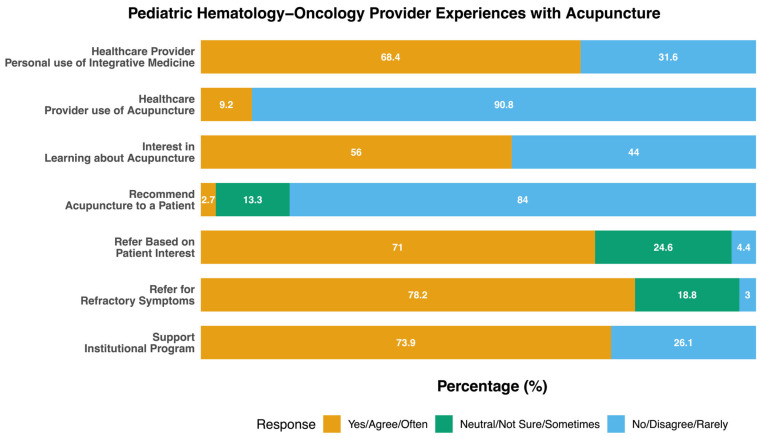
Pediatric hematology–oncology provider’s beliefs about patient access to and personal experiences with acupuncture.

In total, *n* = 78 PHO trained physicians and advanced practice providers (nurse practitioner, physician assistant, certified nurse anesthetist) participated in this needs assessment, with a response rate of 29%. All participants worked at a pediatric institution dedicated solely to caring for children with cancer and blood disorders. Most providers spent >50% of their time in the clinical care of PHO patients and many were in their first 5 years of PHO practice (32.1%, *n* = 25; Table 1). The median practitioner age was 41 years and 67.9% (*n* = 53) self-reported as female (Table 1). Practitioners were primarily white, non-Hispanic, and the majority practiced a form of Christianity (Table 1). Most providers noted personal use of integrative modalities (68.4%, *n* = 52; Figure 1), but only 9.2% (*n* = 7) had received acupuncture themselves in the preceding 3 months (Figure 1).

Most participants were interested in learning more about acupuncture (56.0%, *n* = 42; Figure 1) and said they rarely or never recommend acupuncture to their patients (22.7%, *n* = 17 and 61.3%, *n* = 46, respectively; Figure 1). However, most providers said they would be willing to refer an interested patient for acupuncture if a reputable provider was identified (strongly agree 21.7%, *n* = 15 agree 49.3%, *n* = 34; Figure 1). Despite these gaps, 73.9% (*n* = 51) of participants noted that they would support institutional development of an acupuncture program for PHO patients (Figure 1). When asked about their beliefs surrounding integrative modalities, almost 10% (*n* = 7) noted that most complementary therapies benefit patients due to placebo effect while the majority disagreed (51.4%, *n* = 37; Table 2). More than half of participants (60.6%, *n* = 44) noted that providers trained in both Eastern and Western medicine techniques may have improved patient satisfaction (Table 2).

When asked about the safety of acupuncture in children with hematologic and oncologic disorders, over half (52.2%, *n* = 36) noted that a platelet count of 20,000 or more would be appropriate for receipt of acupuncture while 42% (*n* = 29) were unsure (Table 2). In terms of absolute neutrophil count (ANC), most providers felt that a threshold of greater than or equal to 500 would be adequate to receive acupuncture (16.2%, *n* = 11 strongly agree; 38.2%, *n* = 26 agree; 35.3%, *n* = 24 neutral; Table 2). Almost two-thirds noted that acupuncture could improve their patient’s quality of life (66.7%, *n* = 52; Table 2).

**Figure 2:**
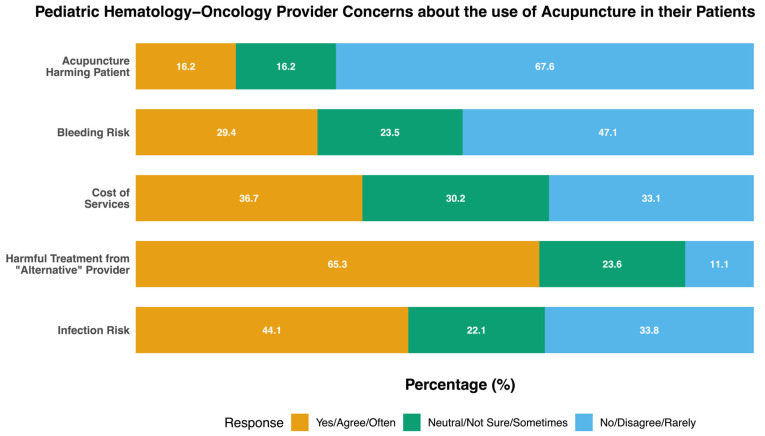
Pediatric hematology–oncology Provider concerns regarding the use of acupuncture in their patients.

Greater than two-thirds of participants expressed that they were not worried overall about harm to their patients through acupuncture (67.6%, *n* = 46; Figure 2). The majority did indicate, however, that they were skeptical of “alternative” healthcare providers causing harm to their patients (65.3% agree or strongly agree, *n* = 47; Figure 2). Participants who endorsed worries about acupuncture for their PHO patients were concerned about risks of bleeding (29.4%, *n* = 20 agree or strongly agree) and infection (44.1%, *n* = 25 agree or strongly agree; Figure 2).

## 4. Discussion

The aim of this study was to understand PHO provider opinions, underlying beliefs, and potential worries regarding the use of acupuncture for their patients. Acupuncture is a form of non-pharmacological symptom management commonly integrated into adult oncology care with a robust evidence base for management of pain, hot flashes, and chemotherapy-induced nausea and vomiting [5,6,7]. Children suffer from similar symptoms during their cancer-directed therapy, yet acupuncture is less well integrated into pediatric cancer care. This needs assessment serves to capture how PHO physicians and APPs think about incorporation of acupuncture into their patient’s care and to better define potential barriers and facilitators to the incorporation of acupuncture for PHO patients.

Participants in this study noted that they use integrative modalities commonly in their own personal healthcare and wellness, but most of this cohort had not recently undergone acupuncture, making it a potentially less-familiar modality. It would be interesting to understand if participants who have other direct experiences with acupuncture, including observation, any previous personal use of acupuncture (rather than within the last 3 months), or use by friends or family members more often discuss acupuncture as an option with patients. Previous work has noted that providers who personally use integrative modalities are more likely to recommend them for patients [17]. Our data echoes this for all integrative modalities; however, PHO provider personal use of acupuncture was uncommon.

Participants also noted they do not routinely recommend acupuncture for their pediatric cancer patients; however, the vast majority were willing to refer an interested patient or family to a reputable acupuncture provider, particularly those with refractory symptoms. This willingness speaks to the high symptom burden experienced by most pediatric cancer patients and the extensive need for therapies that do not add additional side effects. For example, many anti-emetics have the unfortunate side effect of drowsiness, but combining medical therapy with acupuncture would not “double up” on this specific, unwanted result. Overall, it can be interpreted that providers were open to the use of acupuncture for their patients, but their current practice was not to encourage use. It is unclear if the lack of active recommendation is due to bias against the practice versus a lack of knowledge or familiarity with the evidence for use.

This needs assessment was undertaken at a time when there was no access to pediatric acupuncture at our institution, yet an integrative health and medicine program was being developed [19]. It is possible that the PHO providers’ lack of experience with and/or access to a known acupuncture provider limited their willingness to consider or recommend acupuncture for their patients. In fact, in a recent survey of pediatric pain management clinics, it was documented that the Southern United States, where this data was collected, has less access to complementary and integrative therapies and therefore acupuncture may not be top of mind for providers at our center [20].

There are no guidelines for the use of acupuncture in children, much less in children undergoing cancer-directed therapy. In fact, there are no evidence-based rubrics, only expert opinions, in terms of platelet or neutrophil counts, to guide safety for adult patients [21,22]. Oncology acupuncture practice requires that practitioners recognize the inherent risks for patients with chemotherapy-associated cytopenias leading to a potentially higher risk of bleeding and infection [23,24]. About half of the participants in this study believe that a platelet count of >20,000 is reasonable for pediatric acupuncture. We chose to specifically ask providers about this value, as our standard practice at the time was to transfuse platelets for a count less than 20,000. Newer adult recommendations are even more platelet transfusion-sparing, noting that there is moderate-quality data indicating that transfusion of platelets can be held for non-bleeding patients receiving chemotherapy for counts <10,000 [25]. At our institution, lumbar punctures with intrathecal chemotherapy are routinely performed with a 20,000/uL platelet requirement, so it is somewhat surprising that almost half of the participants were “neutral” in their opinion about the safety of acupuncture at this platelet threshold as acupuncture is much less invasive. This neutrality in opinion may be due to the lack of clear guidelines or, more likely, a gap in provider education about the safety of acupuncture. In fact, most acupuncture needles are between 30 and 40 gauge, making them much thinner than the needles used for accessing a port-a-cath or performing lumbar punctures or bone marrow aspirations. In addition to their thinness, acupuncture needles are solid bore and inserted into the subcutaneous tissue rather than intravascularly or intrathecally, meaning they cause less tissue damage and have a lower infection risk. Several large cohorts of adult patients with thrombocytopenia, even with platelet counts < 10,000, show the actual risk of significant bleeding approaches zero [26]. There are data in pediatric cancer patients, although small in number, showing that even with severe thrombocytopenia (platelet count <20,000), the risk of bleeding with acupuncture is minimal [27]. More safety data specifically in the pediatric cancer population with thrombocytopenia may ameliorate fear surrounding the bleeding risk with this procedure. In our recently published work, we had no reported safety events during the first 18 months of acupuncture therapy at our institution using parameters of minimum platelet count of 20,000 and an ANC of 500 for patients receiving treatment with needles [18].

Infection risk is always a prominent concern for PHO providers and cancer patients alike. In this cohort, about half of providers agreed that acupuncture would be safe for patients with an ANC > 500, while the rest were neutral or disagreed with this belief. Localized or distant infection is always possible when there is a break in the skin barrier, but acupuncture performed by a knowledgeable practitioner with sterile, single-use, solid bore needles poses minimal risk. In fact, in a large cohort of 229,000 patients receiving over 2.2 million acupuncture sessions, the overall risk of adverse events of acupuncture requiring treatment or follow up was 2.2% [28]. Thirty-one patients had documentation of local infection (with 24 of 31 requiring treatment), meaning that the risk of localized infection in this cohort was 0.014%.

Anecdotally, pediatric providers may worry about “needle phobia” and limit referrals based on their perceptions. However, in terms of acceptability, pediatric patients referred for acupuncture for a variety of pain symptoms and who underwent acupuncture treatment, when contacted after, noted that acupuncture was “pleasant” (67% children/60% parents), and most (70% children/59% parents) felt the treatment had improved their symptoms [29]. Skilled acupuncturists can work with anxious pediatric patients to trial needles and/or offer acupressure alone. Our institutional practice is to remind patients that they determine what their treatment will look like in terms of use of needles, numbers of needles, and needle site preferences. Children with cancer should never feel obligated to receive acupuncture treatment but should be offered consultation with a trained provider when refractory symptoms that acupuncture may benefit are present.

For any intervention, such as the decision to utilize acupuncture for pediatric oncology patients, the associated risks and benefits must be carefully weighed. Kemper et al. developed a “four box method” to weigh the potential for efficacy and safety for common integrative interventions that includes consideration of medical indications, patient preferences, and quality of life features [30]. Interventions that are definitively safe and efficacious should always be recommended, and those that are definitely unsafe should never be supported. Based on the limited data available, acupuncture is potentially beneficial for pediatric symptom management and appears to display overall safety. For this reason, it is an acceptable form of therapy to offer to pediatric patients with refractory symptoms. Well-designed, randomized studies are needed to document symptom-specific efficacy evidence for pediatric oncology acupuncture; however, funding for these studies is limited and design can be challenging based on the use of controls, placebo, and/or so-called “sham acupuncture” [31].

The strengths of this study are that the surveyed participants were all PHO trained and work solely with PHO patients, making the numbers reasonably robust for a specific provider group—most single centers lack this many oncology-specific providers. In addition, there is a lack of data from a pediatric provider perspective about acupuncture, which we hope to begin to fill. The last PHO survey regarding IM collected was published over 15 years ago, and attitudes have changed about IM in the public and medical circles as well [16]. Finally, when this survey was distributed, participants practiced in a center without acupuncture resources, but over the course of program development, comfort level with acupuncture has increased and access has expanded significantly [18]. We acknowledge this study has limitations including data from a single, albeit large, cancer center and a relatively low, yet literature-consistent, response rate from physicians [32]. A recent study of physician participation showed that less than one-fourth of physicians even open an email survey, much less complete it [33]. That said, our population of providers may have been motivated to participate by their personal opinions, either positive or negative, regarding acupuncture or complementary medicine use, which introduces bias to our results. There are no other similar physician-facing studies of pediatric oncology acupuncture to compare our findings with directly. Finally, this survey did not require open-ended questions surrounding barriers to acupuncture. The opportunity for insight in free-form response was included but the response was exceedingly low, so qualitative explanation of identified barriers was lacking. We hope to mitigate some of these limitations in further provider-facing surveys addressed to a larger, national sample of pediatric oncology providers.

## 5. Conclusions

Children with cancer deserve robust symptom management during therapy and should have access to all modalities that are safe and efficacious for optimal care. Acupuncture has been successfully incorporated into adult oncology symptom guidelines and cancer centers, yet use in pediatric patients is less common. PHO providers would benefit from education surrounding the use of acupuncture for their patients and are overall willing to refer for acupuncture despite some concerns regarding bleeding and infection. Rigorous data for the use of acupuncture for symptom management in children with cancer are needed, and large, well-designed trials to document safety, acceptability, and symptom specific efficacy are required.

## Figures and Tables

**Table 1:** Participant demographics of pediatric hematology–oncology providers.

	*n* = 78	*n* (%)
Occupation	Attending	38 (48.7)
	Fellow	7 (9.0)
	Nurse Practitioner	28 (35.9)
	Physician Assistant	2 (2.6)
	Nurse Anesthetist	3 (3.8)
Clinical time	1–25%	6 (7.7)
	26–50%	14 (17.9)
	51–75%	18 (23.1)
	76–100%	40 (51.3)
Years in PHO practice	1–5 years	25 (32.1)
	6–10 years	14 (17.9)
	11–15 years	11 (14.1)
	16–20 years	11 (14.1)
	>20 years	13 (16.7)
	Missing	4 (5.1)
Age (years)	Median (Q1–Q3)	41 (36–53)
Gender	Male	23 (29.5)
	Female	53 (67.9)
	Missing	2 (2.6)
Race	White	64 (82.1)
	Black	2 (2.5)
	Asian	8 (10.3)
	Other	1 (1.3)
	Missing	3 (3.8)
Ethnicity	Hispanic/Latinx	3 (3.8)
	Not Hispanic/Latinx	71 (91.1)
	Prefer not to answer	4 (5.1)
Religion	Protestant Christian	36 (46.2)
	Roman Catholic	14 (17.9)
	Atheist or Agnostic	9 (11.5)
	None/Missing	12 (15.3)
	Other *	7 (9.1)
Spirituality	Not Spiritual	5 (6.4)
	Slightly Spiritual	15 (19.2)
	Moderately Spiritual	28 (36.0)
	Very Spiritual	27 (34.6)
	Missing	3 (3.8)

PHO = Pediatric Hematology–Oncology. * Other religions = Eastern Orthodox, Buddhism, Hinduism, Judaism, Islam.

**Table 2:** Pediatric hematology–oncology provider’s underlying health beliefs that may influence beliefs about acupuncture.

		*n* (%)
Effects of complementary therapies are usually the result of the placebo effect (*n* = 72) *	Strongly agree	1 (1.4)
	Agree	6 (8.3)
	Neutral	28 (38.9)
	Disagree	35 (48.6)
	Strongly disagree	2 (2.8)
Strong patient–provider relationship leads to improved outcomes (*n* = 72) *	Strongly agree	42 (58.3)
	Agree	25 (34.7)
	Neutral	4 (5.6)
	Disagree	1 (1.4)
	Strongly disagree	0
A patient’s innate healing capacity often determines the outcome regardless of treatment choices	Strongly agree	10 (14.1)
(*n* = 71) *	Agree	21 (29.6)
	Neutral	26 (36.6)
	Disagree	10 (14.1)
	Strongly disagree	4 (5.6)
Physicians with knowledge of multiple medical systems (Eastern + Western) have improved patient satisfaction (*n* = 71) *	Strongly agree	7 (9.9)
	Agree	36 (50.7)
	Neutral	25 (35.2)
	Disagree	2 (2.8)
	Strongly disagree	1 (1.4)
Acupuncture is safe for PHO patients with platelet counts >20,000 (*n* = 69) *	Strongly agree	10 (14.5)
	Agree	26 (37.7)
	Neutral	29 (42.0)
	Disagree	3 (4.3)
	Strongly disagree	1 (1.4)
Acupuncture for PHO patients with ANC > 500 is safe (*n* = 68) *	Strongly agree	11 (16.2)
	Agree	26 (38.2)
	Neutral	24 (35.3)
	Disagree	5 (7.4)
	Strongly disagree	2 (2.9)
I think acupuncture may improve my patient’s quality of life (*n* = 69) *	Yes	52 (66.7)
	No	2 (2.6)
	I’m not sure	15 (19.2)

ANC = absolute neutrophil count. * Missing answers were excluded leading to a varied *n* in each group as noted in each row.

## Data Availability

Data is contained within the article or Supplementary Materials.

## Supplementary Materials

The following supporting information can be downloaded at https://www.mdpi.com/article/10.3390/children12080961/s1: Table S1: Differences between physician and advanced practice provider beliefs.

## Abbreviations

The following abbreviations are used in this manuscript:

APP	Advanced Practice Provider
ANC	Absolute Neutrophil Count
CINV	Chemotherapy-Induced Nausea/Vomiting
PHO	Pediatric Hematology–Oncology
IM	Integrative Medicine
IMM	Integrative Medicine Modalities
