# Supplementary material for: Pediatric Hematology–Oncology Provider Attitudes and Beliefs About the Use of Acupuncture for Their Patients
Source: Children (Basel). 2025 Jul 22;12(8):961. doi: 10.3390/children12080961 (PMC12384243; doi:10.3390/children12080961)
Supplement: Supplementary file 1 [file children-12-00961-s001.zip › children-3746778-supplementary.pdf]

TABLE S1 Differences between physician and advanced practice provider beliefs

Questions	Q1. Occupation[a]			Test	P
	Overall (N=73)	Attending & Fellows (N=44)	APP (N=29)		
Do you currently utilize any integrative modalities for your own health and wellness?				Chi-square	0.558
Yes	50 (68.5%)	29 (65.9%)	21 (72.4%)		.
No	23 (31.5%)	15 (34.1%)	8 (27.6%)		.
I feel confident in my ability to answer patient/family questions about integrative therapies in pediatric cancer care				Chi-square	0.472
Strongly Agree/Agree	18 (24.7%)	13 (29.5%)	5 (17.2%)		.
Neutral	15 (20.5%)	8 (18.2%)	7 (24.1%)		.
Strongly Disagree/Disagree	40 (54.8%)	23 (52.3%)	17 (58.6%)		.
I would like to learn more about integrative health strategies for pediatric oncology patients				Fisher	0.574
Strongly Agree/Agree	61 (83.6%)	35 (79.5%)	26 (89.7%)		.
Neutral	7 (9.6%)	5 (11.4%)	2 (6.9%)		.
Strongly Disagree/Disagree	5 (6.8%)	4 (9.1%)	1 (3.4%)		.
How many of your patients talk with you about complementary therapies?				Fisher	0.481
0%	6 (8.2%)	4 (9.1%)	2 (6.9%)		.
1-25%	41 (56.2%)	26 (59.1%)	15 (51.7%)		.

Questions	Q1. Occupation[a]			Test	P
	Overall (N=73)	Attending & Fellows (N=44)	APP (N=29)		
26-50%	19 (26.0%)	12 (27.3%)	7 (24.1%)		.
51-75%	6 (8.2%)	2 (4.5%)	4 (13.8%)		.
76-100%	1 (1.4%)	0 (0%)	1 (3.4%)		.

[a] Two missing data points and three “other” in occupation were excluded (N=73).
APP=advanced practice provider, here defined as nurse practitioners AND physicians’ assistants
